# Bacteriologically confirmed extra pulmonary tuberculosis and treatment outcome of patients consulted and treated under program conditions in the littoral region of Cameroon

**DOI:** 10.1186/s12890-018-0770-x

**Published:** 2019-01-17

**Authors:** Teyim Pride Mbuh, Irene Ane-Anyangwe, Wandji Adeline, Benjamin D. Thumamo Pokam, Henry Dilonga Meriki, Wilfred Fon Mbacham

**Affiliations:** 1 Department of Microbiology and Parasitology, Faculty of Science, University of Buea, Buea, Cameroon; 2 Department of Medical Laboratory Science, Faculty Health Sciences, University of Buea, Buea, Cameroon; 3 Tuberculosis Reference Laboratory, Douala, Littoral Region Cameroon; 4 Laboratory for Public Health Research Biotechnologies, Biotechnology Centre, University of Yaoundé, Yaoundé, Cameroon

**Keywords:** Gene xpert MTB/RIF, Extra pulmonary TB, Treatment outcome, Littoral region Cameroon

## Abstract

**Background:**

Extra-pulmonary tuberculosis (EPTB) is defined as any bacteriologically confirmed or clinically diagnosed case of TB involving organs other than the lungs. It is frequently a diagnostic and therapeutic challenge with paucity of data available. The aim of this study was to assess the prevalence of bacteriologically confirmed EPTB; to determine the most affected organs and to evaluate the therapeutic outcome of EPTB patients treated under program conditions in the littoral region of Cameroon.

**Methods:**

A descriptive cross-sectional laboratory-based epidemiological survey was conducted from January 2016 to December 2017 and 109 specimens from 15 of the 39 diagnosis and treatment centers in the littoral region were obtained.

Two diagnostic methods (Gene Xpert MTB and culture (LJ and MGIT) were used for EPTB diagnosis. Determine HIV1/2 and SD Biolinewere used for HIV diagnosis. Confirmed EPTB cases were treated following the national tuberculosis guide.

**Results:**

The prevalence of bacteriologically confirmed EPTB was 41.3% (45). All 45 cases were sensitive to rifampicin. Males were predominately more infected [26 (57.8%)] likewise the age group 31–45 years with 15 (33.3%) cases. The overall prevalence for HIV was 33.6% (36). HIV infection was present in 28.9% (13) of patients with EPTB. The most affected sites with EPTB were: Lymph nodes (66.5%), pleural cavity (15.6%), abdominal organs (11.1%), neuromeningeal (2.2%), joints (2.2%) and heart (2.2%). Overall, 84.4% of the study participants had a therapeutic success with males responding better 57.9% (*p* = 0.442). Therapeutic success was better (71.7%) in HIV negative EPTB patients (*p* = 0.787).

**Conclusion:**

The prevalence of bacteriologically confirmed EPTB patients treated under program conditions in the littoral region of Cameroon is high with a therapeutic success of 84.4% and the lymph nodes is the most affected site.

## Background

Tuberculosis (TB) is a leading cause of morbidity and mortality worldwide, accounting for about 9.6 million new cases and 1.5 million deaths annually [1].

Pulmonary TB represents about 70% of all cases of TB and is the most contagious form TB and remains the main target for TB control [2, 3]. Extra-pulmonary tuberculosis (EPTB) defined as any bacteriologically confirmed or clinically diagnosed case of TB involving organs other than the lungs. The description of a form of EPTB is a function of its location affecting the pleura, lymph nodes, abdomen, genitourinary tract, skin, joints and bones, meninges.

EPTB represents 15 to 30% of all forms of tuberculosis [4, 5], and is frequently a diagnostic and therapeutic challenge. It is a common opportunistic infection in people living with HIV/AIDS and other immunocompromised states [6]. The immune suppression status of these persons results in dissemination of the bacteria form the lungs to other organs. There is a paucity of data on bacteriological diagnosis and therapeutic evaluation on EPTB in Cameroon.

The objective of this study was to assess the prevalence of bacteriologically confirmed EPTB; to determine the most affected organs and to evaluate the therapeutic outcome of EPTB patients treated under program conditions in the littoral region of Cameroon.

## Methods

We conducted a descriptive cross-sectional hospital-based epidemiological survey from January 2016 to December 2017. A non-probabilistic accidental sampling method was used to obtain 145 non sputum specimens and 145 venous blood in dry tubes from 80 male and 65 female participants suspected for EPTB and consulting 15 of the 39 diagnostic and treatment centers (DTCs) in the Littoral region of Cameroon. The littoral region was chosen because it harbours about 20% of all TB diagnosed cases in Cameroon and also because it is the economic head quarter of Cameroon and Douala the regional head quarter is the largest city in Cameroon with a population of 1,338,082 people. It harbors many referral diagnostic and treatment institutions that pulls many persons from the regions in search for better health service. A total of 36 participants were rejected due to incomplete request forms. 109 (57 male and 52 female) participants were finally retained and analyzed for this study. Verbal Consent of participant was sort during clinical consultation. For participants younger than 15 years; consent was obtained from parent/legal representative. Authorizations for this study were obtained from the permanent secretary for national tuberculosis control program and ethical clearance was obtained from the University of Douala’s ethical review board.

EPTB specimens (plural fluid, cerebrospinal fluid, synovial fluid, ascites and lymph node aspirate) obtained from participants were aliquoted and analyzed on Gene Xpert MTB/RIF. This system integrates and automates sample processing, nucleic acid amplification, and detection of the target sequences. The primers in the XpertMTB/RIF assay amplify a portion of the *rpoB* gene containing the 81 base pair “core” region. The probes are able to differentiate between the conserved wild-type sequence and mutations in the core region that are associated with rifampicin resistance. The aliquoted specimen was decontaminated using 3% NaOH and the pellet inoculated on MGIT and grown in BACTEC 960 for 42 days. Positive MGIT samples were further subjected to Gene Xpert MTB/RIF for confirmation and Rifampicin drug susceptibility testing. A positive diagnosis for EPTB was declared when direct Gene XpertMTB/RIF and or culture results were positive.

Bacteriologically confirmed EPTB cases were treated following the national tuberculosis guide which recommended that new cases should be treated for 6 months consisting of 2 months of daily rifampicin (R), isoniazide (H), pyrazinamide (Z) and ethambutol (E) (intensive phase), followed by 4 months of daily RH (2RHZE/4RH). Retreatment cases [relapses, treatment failure cases, defaulters) should be treated for 2 month with RHZE and streptomycin (S), and a third month without S (intensive phase), followed by 5 months of RHE daily (2RHZES/1RHZE/5RHE). At the end of treatment, treatment outcome was categorized as cured, completed treatment, lost to follow up, failure and died based on the standard criteria [7].

HIV screening was done by testing serum from coagulated blood collected in dry tubes on Determine HIV *1/2* strip*test*. Positive samples were confirmed on rapid SD BiolineHIV 1/2 3.0 cassettes.

Data was analyzed using SPSS version 16.0 software (Statistical Package for Social Science). Percentage accuracy, sensitivity, specitivity, positive predictive values, and negative predictive values were manually calculated as follows:

- (%) Accuracy = number of correct results / total number of results X 100,
- (%) Sensitivity = number of true positive results/ number of true-positive plus false-negative results X 100,
- (%) Specificity = number of true negative results/ number of true-negative plus false positive results X 100.
- (%) Positive Predictive Value = number of true-positive results/ number of true-positive plus false-positive results X 100.
- (%) Negative Predictive Value = number of true negative results / number of true-negative results plus false negative results X 100; and statistically significant was set at *p* < 0.05.

## Results

Table 1 shows the socio-demographic characteristics of 109 study participants. The median age of study participant was 40.0 ± 19.23 years with females being relatively younger (35.5 ± 20.0 years) than their male counterparts 45.0 ± 18.3 years). Thirty six (33.6%) of the study population were infected with HIV-1. The prevalence of bacteriologically confirmed EPTB in the study was 45 (41.3%), and all the subjects were sensitive to rifampicin. Of the 45 cases, a male predominance [26 (57.8%)] was observed (*P* = 0.336). The EPTB/HIV co-infection rate was 13 (28.9%). The most affected age group was that of 35–51 years with 15 (33.3%) cases recorded followed by that of 18–34 years with 14 (31.1%). Those who had no treatment history for TB represented 66.6% of the study population as illustrated on Table 2.

**Table 1:** Socio-demographic characteristics of study participants

Characteristics	Category	Frequency	Percentage
Age range (years)	< 18	10	9.2
	18–34	31	28.4
	35–51	39	35.8
	> 51	29	26.6
Gender	Female	52	47.7
	Male	57	52.3
Treatment History	Yes	34	31.2
	No	75	68.8
HIV	Positive	36	33.0
	Negative	71	65.1
Patient type	New	75	68.8
	Relapse	27	24.8
	Return after loss to follow up	4	3.7
	Failure	3	2.8

**Table 2:** Distribution extra pulmonary tuberculosis (EPTB) according to sociodemographic and clinical characteristics of study participants

Characteristics of participant	Category			EPTB results		
		N	Positive (%)	Negative (%)	(95% CI;)	*p*-value
Age range (years)	< 18	10	7 (15.6)	3 (4.7)	35.8–97.4	0.244
	18–34	31	14 (31.1)	19 (26.6)	27.4–60.8	
	35–51	33	15 (33.3)	24 (37.5)	25.6–59.3	
	> 51	29	9 (20.0)	20 (31.2)	14.6–45.9	
Gender	Male	57	26 (45.6)	31 (54.4)	32.7–58.5	0.336
	Female	52	19 (36.5)	33 (63.5)	23.5–49.6	
HIV	Positive	36	13 (28.9)	23 (37.1)	23.6–57.6	.375
	Negative	71	32 (71.1)	39 (62.9)	39.1–62.3	
Treatment History	Yes	34	15 (44.1)	19 (55.9)	27.4–60.8	0.421
	No	75	30 (40.0)	45 (60.0)	28.9–51.1	
Patient type	New	75	30 (40.0)	45 (60.0)	28.9–51.1	0.706
	Relapse	27	12 (44.4)	15 (55.6)	25.7–63.2	
	Return after loss to follow up	4	1 (25.0)	3 (75.0)	17.4–67.4	
	Failure	3	2 (66.7)	1 (33.3)	13.3–20.2	

**Fig. 1:**
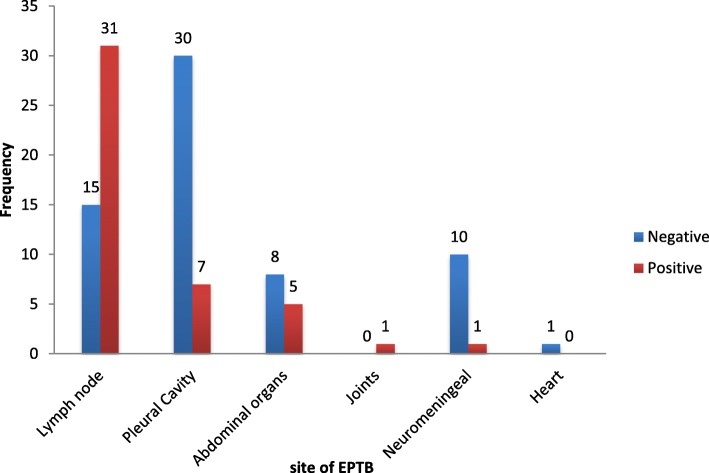
Prevalence of extra pulmonary tuberculosis according to the organ sites affected

The most affected site were the lymph nodes 31/109 (68.9%) and there was a statistically significant different between the positivity of lymph node aspirates and all other EPTB specimens tested [*p* = 0.001, CI 55.4–82.4, OR=] this was closely followed by the pleural cavity 7 (15.6%) as illustrated on Fig. 1. Based on both diagnostic methods used in our study, the overall prevalence of extra pulmonary tuberculosis was 45 (41.3%). There was a positive discordance in 7 cases, and negative discordance in 4 cases as illustrated in Table 3.

**Table 3:** Comparison of diagnostic efficacy of Extra pulmonary using Gene Xpert and culture

Specimen type	N	Culture	Gene Xpert Negative (%)	Gene Xpert Positive (%)	Accuracy (95% CI)	Sensitivity (95% CI)	Specificity (95% CI)	PPV (95% CI)	NPV (95% CI)
Lymph Node Aspirates	46	Negative	15 (32.6)	4 (8.7)	84.8	88.9	78.9	85.7	83.3
		Positive	3 (6.5)	24 (52.2)	74.4–95.2	77.1–100	60.6–97.3	72.8–98.7	66.1–98.2
Pleural Fluid	37	Negative	30 (81.2)	2 (5.4)	91.9	0.8	93.8	66.7	96.8
		Positive	1 (2.7)	4 (10.8)	83.1–100	44.9–98.2	85.4–100	28.9–100	90.5–100
Ascites	13	Negative	8 (61.5)	1 (7.7)	92.3	100	88.9	80	100
		Positive	0 (0)	4 (3.08)	77.8–100	100–100	68.4–100	44.9–100	100–100
Synovial Fluid	2	Negative	0 (0)	1 (100)	50	100		50	
		Positive	0 (0)	1 (50)	19.3–19.3	100–100		19.3–19.3	
Cerebrospinal fluid	11	Negative	10 (90.9)	0 (0)	100	100	100	100	100
		Positive	0 (0)	1 (9.1)	100–100	100–100	100–100	100–100	100–100
Pericardial Fluid	1	Negative	1 (100)	0 (0)	100		100		100
		Positive	0 (0)	0 (0)	100–100		100–100		100–100
Total specimens	109	Negative	64 (58.7)	7 (6.4)	89.0	89.2	90.15	82.5	94.1
		Positive	4 (3.7)	34 (31.2)	84.3–95	79.7–99.2	83.2–97.1	71.4–94.4	88.5–99.7

*CI* confidence interval, *PPV* positive predictive value, *NPV* Negative predictive value

By observing the treatment outcome of the studied participants, we found that 38 (84.4%) cases have completed the treatment course. Five (11.1%) cases died before completing the course and were all EPTB/HIV co-infected. Two (4.2%) cases were loss to follow up and no defaulter or relapse cases were recorded.

Overall, 84.4% of these participants had a therapeutic success with males responding better 22 (57.9%) as oppose to females16 (42.1%) (*p* = 0.442). Therapeutic success [27(71.7%)] was better in HIV negative EPTB patients compared to HIV positive [11(28.1)] patients (*p* = 0.787). With respect to site, treatment completion rate was observed as follows: lymph node 25 (83.3%), pleural effusion 7 (100%), abdominal organs 4(80%), joints 1(100%), neuromeningeal 1(100%) and the heart 0(0%) as shown on Table 4.

**Table 4:** Therapeutic outcome of EPTB with respect of site of infection and HIV status

site of infection	HIV Status	Died (%)	Complete (%)	Loss to Follow up (%)
Lymph node	Negative	0 (0)	17 (68)	1 (100)
	Positive	4 (100)	8 (32)	0 (0)
7 Pleural Cavity	Negative	0 (0)	6 (85.7)	0 (0)
	Positive	0 (0)	1 (14.3)	0 (0)
5 Abdominal organs	Negative	0 (0)	3 (75)	1 (100)
	Positive	0 (0)	1 (25)	0 (0)
1 Joints	Negative	0 (0)	0 (0)	0 (0)
	Positive	0 (0)	1 (100)	0 (0)
1 Neuromeningeal	Negative	0 (0)	1 (100)	0()
	Positive	0 (0)	0 (0)	0 (0)
1 Heart	Negative	0 (0)	0 (0)	0 (0)
	Positive	1 (100)	0 (0)	0 (0)
All Sites	Negative	1 (20)	27 (71.7)	2 (100)
	Positive	4 (80)	11 (28.1)	0 (0)

## Discussion and conclusion

This study was aimed at determining the prevalence of bacteriologically confirmed EPTB; to determine the most affected organs and to evaluate the treatment outcome of EPTB patients. Though the sampling method was non-probabilistic (not give all those visiting the study sites equal chances of participating in our study), we still thing these findings are of epidemiological importance.

The prevalence of bacteriologically confirm EPTB in this study was high (41.3%). This finding is similar to those of Luma [8], were they found a prevalence of EPTB to be 42.9% amongst patients registered for anti-TB treatment in the general hospital of Douala in the same country. This was however higher than the 23.2% reported by Yone et al., [9] in Yaoundé-Cameroon. This high prevalence of EPTB reported in our study could be partially justified by the elevated (33.6%) prevalence of HIV in our study participant. HIV has be known to facilitates the dissemination of *Mycobacterium tuberculosis* out of the lungs and the reactivation of infection in extra-pulmonary organs [10]. Bacteriological confirmation of EPTB is a very challenging issue in most national tuberculosis programs because of the pauci-bacillary nature of the disease, the apportioning of the sample for various diagnostic tests resulting in non-uniform distribution of microorganisms, the difficulty to obtain an adequate sample, the variable clinical presentation, and need for invasive procedures to secure appropriate sample [11], lack of laboratory facilities in the resource-limited settings and the lack of an efficient sample processing technique universally applicable on all types of extra-pulmonary samples [12]. All these limitations cause poor contribution of bacteriological techniques in the establishment of EPTB diagnosis [13].The male gender was predominantly more represented than the female gender. Those aged from 35 to 51 years old were also the most infected. These findings are similar to those of Kiran [14] in Morocco who found a significant male predilection (59.3%). The relative higher incidence in males could be attributed to more exposure of the male gender to the external environment for their jobs especially as they are the principal bread winners in resource limited counties.

We found that lymph node involvement was the most common site of infection. This was different from recent studies [8, 15] where they found that the pleural and bones/joints were the most common site affected respectively. Our findings where however in line with other studies [15–17], who also reported lymph node involvement as the most affected EPTB site. This findings could be partially justified by the fact that we worked with several health facilities contrary to the Luma’s study who worked in a single health facility [8].

Even though culture is considered the gold standard in TB diagnostics, growth on solid culture media requires four to six weeks. This delay would negatively affect patient care. To overcome this problem, we opted for automated cartridge-based molecular nucleic acid amplification (NAA) techniques, which offer a rapid diagnosis of life-threatening disease such as TB meningitis with a turnaround time of 24 h. This method is said to be very sensitive as it can detect as few as 10 mycobacteria [13]. There was a positive discordance in 7 cases, and negative discordance in 4 cases with MGIT cultures. Other studies [12, 14, 18], however report very varying sensitivity and specificity of molecular nucleic acid amplification techniques in comparison with culture.

EPTB treatment success rate in this study was high and identical to the nationwide pulmonary TB therapeutic success rate of 75 to 84% reported in 2006 to 2015 [19, 20]. This could be attributed to the fact that we assigned a staff to call our patients very regularly to ensure that they all complied to their treatment even when they no longer felt sick; thought this is not always feasible in real life setting. This finding was in line with the stop TB strategy united nations millennium development goals to cure at least 85% of sputum smear-positive TB patients [21].

This study established that treatment success of EPTB patients co-infected with HIV was lower (28.1%) compared to TB-HIV negative patients (71.7%) and this is in line with Atekem, et al [22] in the South West region of Cameroon, but contrary to that of Mekonnenet al. [23] in North Eastern Ethiopia. Therapeutic success in this study was relatively high because we assigned a staff to call our patients very regularly to ensure that they all complied to their treatment even when they no longer felt sick. All the patients who died in this study were HIV positively co-infected, but the numbers were too small for a proper analysis. We thing that these patients may not have been compliant to their treatment and turned to neglect the regimen as they thought having HIV is the end of life. Furthermore, a weaken immune system may justify the death cases recorded in these HIV positive cases.There was no significant different between males and females and across the different age groups.

## Conclusion

The prevalence of bacteriologically confirmed EPTB patients treated under program conditions in the littoral region of Cameroon is high with a therapeutic success of 84.4% and the lymph nodes is the most affected site.

## Abbreviations

CI: Confidence interval
EPTB: Extra-pulmonary tuberculosis
HIV: Human immune deficiency virus
LJ: Lowenstein Jensen media
MGIT: Minimum Growth Inhibition Tube
MTB/RIF: *Mycobacterium tuberculosis* / rifampicin
NPV: Negative predictive value
PPV: Positive predictive value
TB: Tuberculosis
